# Dendrite formation in silicon anodes of lithium-ion batteries

**DOI:** 10.1039/c7ra12690e

**Published:** 2018-01-29

**Authors:** Luis A. Selis, Jorge M. Seminario

**Affiliations:** Department of Chemical Engineering, Department of Electrical and Computer Engineering, Department of Materials Science and Engineering, Texas A&M University College Station TX 77843 USA seminario@tamu.edu +1 979 845-3301

## Abstract

Rechargeable lithium-ion batteries require a vigorous improvement if we want to use them massively for high energy applications. Silicon and metal lithium anodes are excellent alternatives because of their large theoretical capacity when compared to graphite used in practically all rechargeable Li-ion batteries. However, several problems need to be addressed satisfactorily before a major fabrication effort can be launched; for instance, the growth of lithium dendrites is one of the most important to take care due to safety issues. In this work we attempt to predict the mechanism of dendrite growth by simulating possible behaviors of charge distributions in the anode of an already cracked solid electrolyte interphase of a nanobattery, which is under the application of an external field representing the charging of the battery; thus, elucidating the conditions for dendrite growth. The extremely slow drift velocity of the Li-ions of ∼1 mm per hour in a typical commercial Li-ion battery, makes the growth of a dendrite take a few hours; however, once a Li-ion arrives at an active site of the anode, it takes an extremely short time of ∼1 ps to react. This large difference in time-scales allows us to perform the molecular dynamics simulation of the ions at much larger drift velocities, so we can have valuable results in reasonable computational times. The conditions before the growth are assumed and conditions that do not lead to the growth are ignored. We performed molecular dynamics simulations of a pre-lithiated silicon anode with a Li : Si ratio of 21 : 5, corresponding to a fully charged battery. We simulate the dendrite growth by testing a few charge distributions in a nanosized square representing a crack of the solid electrolyte interphase, which is where the electrolyte solution comes into direct contact with the LiSi alloy anode. Depending on the selected charge distributions for such an anode surface, the dendrites grow during the simulation when an external field is applied. We found that dendrites grow when strong deviations of charge distributions take place on the surface of the crack. Results from this work are important in finding ways to constrain lithium dendrite growth using tailored coatings or pre-coatings covering the LiSi alloy anode.

## Abstract 2

Dendrite formation conditions on a SEI crack exposing the silicon anode to the electrolyte during the charging of a LIB.

## Graphical abstract

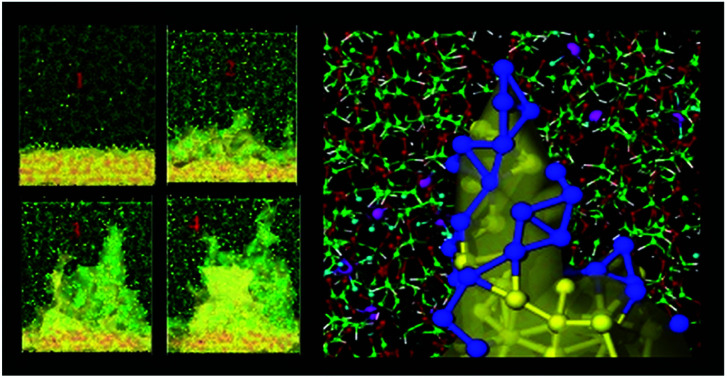

## Introduction

Lithium-ion batteries are devices that transform available electrical energy into chemical energy, so the energy can be transformed back and delivered as electrical energy when needed. Presently, a typical lithium-ion battery (LIB) consists mainly of a graphite anode, a LiCoO_2_ cathode, an electrolyte solution made of ethylene carbonate (EC)^1,2^ with a dissolved LiPF_6_ salt, a separator, and two metallic current collectors. Presently, there are strong efforts to design more powerful, more efficient, and more environmentally compatible materials for batteries.

Silicon and Li-metal have been proposed as promising anode materials for rechargeable LIB^3,4^ because of their high theoretical capacity of 4212 mA h g^−1^ (ref. 5) (for Li_22_Si_5_),^6^ and 3860 mA h g^−1^,^7,8^ respectively. Li-metal has low density, 0.59 g cm^−3^,^9^ and very low absolute electrode potential of 1.40 V that is −3.04 V *vs.* the standard hydrogen electrode,^9,10^ (with an absolute electrode potential of 4.44 V).^11^ These excellent characteristics surpass those of present graphite anodes that have a capacity of 372 mA h g^−1^, a density between 2.09 and 2.23 g cm^−3^, and an electrode potential of ∼0.1 V *vs.* the Li/Li^+^ electrode.^12^ On the other hand, Si is an abundant material, less expensive than graphite,^13^ with a density of 2.33 g cm^−3^,^14^ and an electrode potential very close to the one of Li/Li^+^. These characteristics of lithium-ion batteries have triggered extensive research during the last 45 years and currently have a critical role in meeting the ever-growing demands for higher energy density batteries, targeting applications such as electric vehicles, power electronic devices, and grid energy backups.^15–18^

Due to its low packing factor (0.34), the diamond structure of Si has a large capacity to store Li. Therefore, it yields a large volume expansion of approximately 300% at full lithiation, causing mechanical stresses that produce cracks in the solid electrolyte interphase (SEI), leading to a loss of electrical contact and capacity fading, increased impedance, and thermal runaway, *i.e.*, in practical terms, a general failure of the battery.^19^ Another critical problem with Li-ions is that they may suffer the effect of plating instead of alloying with the Si, triggering the formation of dendrites, which might produce internal short circuits in the battery.^20^ Over the years several investigations have been done on how to prevent the growth of dendrites in a battery,^21–24^ but only a few theoretical studies of this phenomenon have been reported^25,26^ and despite them, the exact mechanisms of growing dendrite are still elusive.^9,27^ As mentioned before, Si anodes swell about 300% and hard SEI such as LiF most likely crack due to such expansion, allowing Li-ions to be reduced and then nucleate into the cracks forming dendrites. There is a lot of recent experimental and computational work being reported on dendrites.^28–38^

One of the causes of dendrites formation in Si anodes may be the rapid nucleation of uneven Li deposition in a small area of the anode surface, especially in regions where the SEI is cracked. In this work we perform MD simulations to model the growth of dendrites on a portion of the anode surface exposing a cracked SEI. Since cracking takes place when the silicon anode is almost or totally lithiated, a previously lithiated silicon anode with a Li : Si ratio of 21 : 5 is used. At this ratio, the lithiated Si already shows metallic properties,^39^ and we simulate the flow of lithium ions on this surface under the effects of an electric field. Thus, this work focuses on elucidating the conditions for dendrite growth, conditions before the growth are assumed, and conditions that do not lead to growth are ignored. This work asks: if dendrites grow at some point, what are the possible conditions that favor such a growth? In the next section, the methodology of the molecular dynamics simulations performed to investigate the behavior of the lithium dendrites is provided, followed by the results and discussion, and ending up with the conclusions.

## Methodology

Classical molecular dynamics (MD) simulations are performed using the Large-Scale Atomic/Molecular Massively Parallel Simulator (LAMMPS) program developed by Plimpton *et al.*^40^ We also use the visual molecular dynamics software (VMD)^41^ and the 3D visualization Open Visualization Tool (OVITO)^42^ to visualize and to perform post-molecular dynamics calculations, as well as the PACKMOL^43,44^ program, specially to build the initial positions of the electrolyte and counterions.

### Force fields

A second nearest-neighbour (2NN) embedded MEAM^45,46^ force field is employed for the silicon lithiated anode structure and its interaction with Li^+^ ions and Li^−^ (Table 1) is given by
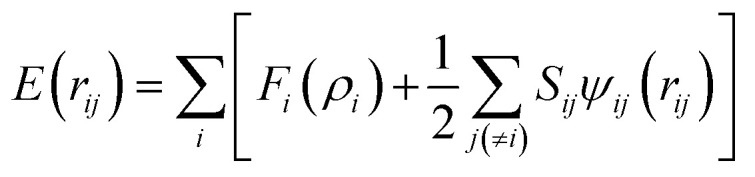
with *F*_*i*_(*ρ*_*i*_) as the embedding function for atom *i* embedded in a background electron density *ρ*_*i*_, *Ψ*_*ij*_(*r*_*ij*_) as the pair potential between atoms *i* and *j* separated by a distance *r*_*ij*_, and *S*_*ij*_ as the screening factor. The quantities used to generate the functions *F*_*i*_ using density functional theory (DFT) are given in Table 1, where *E*_c_ is the cohesive or sublimation energy, *r*_e_ is the equilibrium distance, *r*_c_ is the cutoff radius, *ρ*^Si^_o_/*ρ*^Li^_o_ is the relative density for the silicon with *ρ*^Li^_o_, the background reference electron density, and *C*_max_ and *C*_min_ are the screening parameters.

**Table 1:** MEAM Potential Parameters for Li–Si^45^

Parameter	Value
Lattice	*l*12
*E*_c_ (kcal mol^−1^)	56.499
*r*_e_ (Å)	2.75
*α*	4.1
*d* (Å)	0.1
*ρ* ^Si^_o_/*ρ*^Li^_o_	3
*r*_c_ (Å)	10
*C*_max_ (Li–Li–Si)	2.81
*C*_max_ (Si–Si–Li)	2.2
*C*_max_ (Li–Si–Li)	2.4
*C*_max_ (Li–Si–Si)	2.4
*C*_min_ (Li–Li–Si)	0.55
*C*_min_ (Si–Si–Li)	0.35
*C*_min_ (Li–Si–Li)	0.45
*C*_min_ (Li–Si–Si)	0.45

The reference structure Li_3_Si-type L1_2_^(ref. 45^) with only one type of atoms as first nearest-neighbour and the same type of atoms as second nearest-neighbour is used to evaluate *F*_*i*_(*ρ*_*i*_) and *ψ*_*ij*_. *F*_*i*_(*ρ*_*i*_) = *AE*_c_(*ρ*_*i*_/*ρ*^0^_*i*_)ln(*ρ*_*i*_/*ρ*^0^_*i*_), where *A* is an adjustable parameter. The universal equation of state of Rose *et al.*^47^ is used to calculate the total energy per atom of the reference structure,
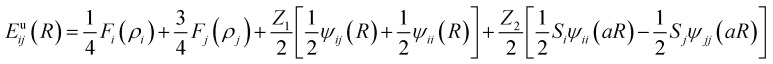
where *S*_*i*_ and *S*_*j*_ are the screening function for the second nearest-neighbour interactions between *i* and *j* atoms, *Z*_1_ and *Z*_2_ are the atomic numbers of first and second nearest neighbors, *R* is the distance between nearest neighbors, and a is the ratio between the first and second nearest-neighbor distance in the reference structure. The pair potential interaction corresponding to the reference structure is given by,
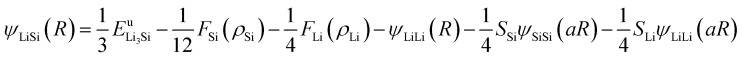


Thus, *ψ*_LiSi_(*R*) is the universal function for a uniform expansion or contraction of the reference structure as a function of nearest neighbor distance *R*. The energy per atom for the reference structure is obtained from the universal equation of state,^48^
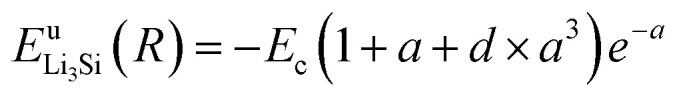
, where 
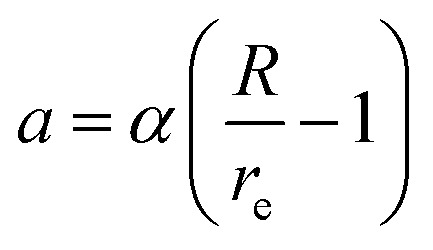
, 
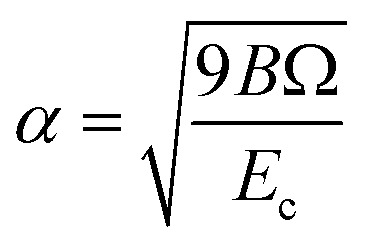
, *d* is an adjustable parameter used to fit the MEAM potential for a LiSi alloy, *B* is the bulk modulus, *r*_e_ is the equilibrium NN distance, and *Ω* is the equilibrium atomic volume. The pair potential interaction between atoms of the same type (*ψ*_SiSi_ and *ψ*_LiLi_) can be computed from the calculations made of the individual elements. Finally, the many body screening function *S*_*ij*_ between atoms *i* and *j* is defined as the product of the screening factor, *S*_*ikj*_, due to all other atoms between *i*, *k*, and *j*. 
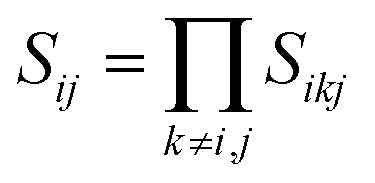
, where 
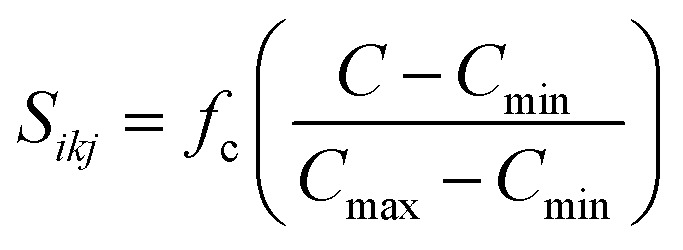
 and *f*_c_ is the cutoff function. The screening parameters *C*_min_ and *C*_max_ are also crucial in determining the interaction range of the alloys.^49^

**Fig. 1:**
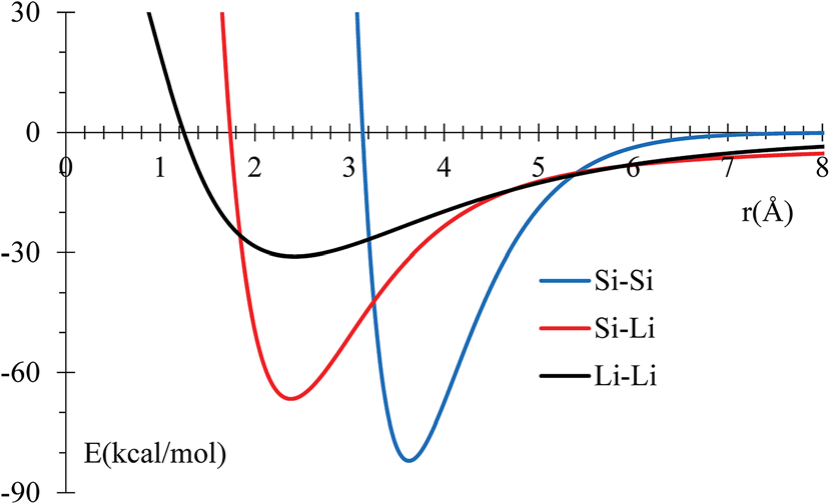
Potential energy profile for Si and Li interactions using MEAM potential. Equilibrium distances are consistent with experimental data (inside parentheses): 3.628 (3.84),^50^ 2.379 (2.355),^51^ and 2.419 (2.672),^52^ for Si–Si, Si–Li, and Li–Li, respectively. All distances are in Å.

MEAM potential interactions Si–Li, Si–Si, Li–Si using only 2 atoms (Fig. 1) in each case, allows us to adjust individual equilibrium distances and binding energies.

Nonbonded interactions in the solvent and between the solvent and LiSi alloy are simulated with a Lennard–Jones (*L*–*J*) potential in conjunction with coulombic parameters (Table 2):
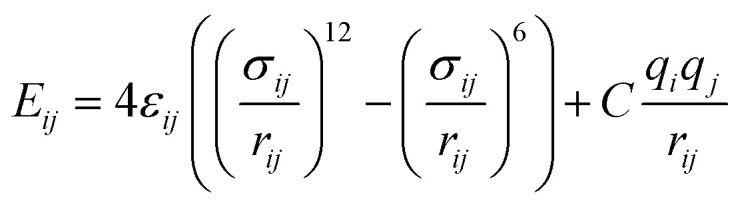
where *r*_*ij*_ is the distance between any intermolecular pair of atoms *i* and *j*, and *ε* and *σ* are the well-depth and zero-energy length, respectively, of the *L*–*J* potential. The equilibrium distances and binding energies can be strongly modified by the coulombic term (Table 2). For nonbonded interactions between atoms, geometric and arithmetic mean approximations are used for *ε* and *σ*, respectively. These *L*–*J* parameters for EC were taken from Masia *et al.*^53^ and for PF_6_^−^ from Jorn *et al.*,^54^ except for the sigma of P that was taken from the UFF of Rappe *et al.*^55^ The Si *L*–*J* parameters were also taken from the UFF of Rappe *et al.*^55^ All charges were taken from Galvez-Aranda *et al.*^56^ who performed B3PW91/6-31G(d) as coded in the program Gaussian-09.^57^ The specific intramolecular force constants for EC were taken from the nonreactive CFF93 all-atom force field of Sun *et al.*;^58^ however, the bond lengths, angles and dihedrals of EC were taken from the B3PW91/6-31G(d) of Galvez-Aranda *et al.*;^56^ in which, this combination of force fields was used successfully in a Li-ion full nanobattery with Si electrodes,^56^ and also with a very similar combined force-field by Kumar *et al.*^59^ in the modelling of Li-diffusion through the electrolyte, obtaining good agreement with experimental results.

**Table 2:** Nonbonded Lennard–Jones and coulombic parameters

Atom	*ε* ^59,60^ (kcal mol^−1^)	*σ* ^59,60^ (Å)	*q* ^59^ (*e*)
Li^+^	0.103	1.442	1.00
F^−^	0.028	2.934	−0.40
P	0.131	3.695	1.39
PF_6_^−^			−1.00
O=	0.210	2.960	−0.60
O–	0.170	3.000	−0.54
C(sp^3^)	0.105	3.750	−0.05
C(sp^2^)	0.066	3.500	1.04
H	0.030	2.500	0.20
Si	0.402	4.295	0.00

Although we use a dissociative force field for the anode, we are not studying the decomposition of EC and thus neither the formation of the SEI. This work focuses on a SEI that is already formed and cracked by the expansion of the Si anode due to its lithiation;^56^ therefore, the use of a nonreactive force field for EC is fully justified. These simulations correspond to a series of events occurring at a fast pace during an overcharging of the battery. Although this requires the application of very high electric fields, we make sure that they are not high enough that they cannot maintain the potential fluctuations in the bulk of the electrolyte, on average, constant; otherwise, it would end up having the electrolyte polarized. The onset of this polarization is actually an indicator of the maximum electric field that can be fully screened at the electrodes, yielding a constant potential in the bulk of the electrolyte that can be used to get reliable results.

On the other hand, a simulation that would cover the performance of a new battery through its life time, passing through the formation of the SEI, and followed by a number of charge–discharge cycles until the plating or the formation of dendrites is really out of the scope of any present realistic and scientifically useful simulation. A reactive force field such as ReaxFF^61^ would be of interest to study the decomposition of EC leading to the formation of the SEI; however, the formation of dendrites that we are simulating takes place after the formation of the SEI and after it is broken by expansion of an almost or fully lithiated Si anode when negligible dissociation of EC takes place. If electrons tunneling from the anode to the electrolyte solution^62,63^ decompose the EC and re-create the SEI, this would be a competing reaction with plating. If SEI formation dominates, that may again control Li deposition and no dendrites would be formed. This should be good although it irreversibly consumes electrolyte and Li-ions available. However, the case where the decomposition of EC is a dominant reaction, is also not the focus of our study of dendrites formation. Nevertheless, the effect of the EC as a solvent is well-represented by the non-reactive force field and will certainly affect Li-ion flow and how dendrites grow. In addition, the competing reaction process when electrons tunnel to the electrolyte and are absorbed by the Li^+^ in the solvent, may deposit Li atoms on the surface of the electrode or even before the Li^+^ reaches the electrode, yielding the nucleation of Li on the surface of the electrode or the production of dead lithium in the solvent, respectively. Certainly, stronger coulombic forces will try to bring the Li-ions to enter the SiLi complex but at full lithiation in the small area of the crack, Li-ions cannot be inserted further, becoming reduced and yielding to dendrite formation.

It is important to mention that simulation and experimental relevant times cannot be compared. They are calculated and measured, respectively, at very different scales so the former is not a prediction or postdiction of the latter. The main events in electrochemistry take place in fractions of picoseconds or so, but time separations between those events on a surface of the size in our simulations may take several minutes. The times we use, ∼0.1 fs, to follow the chemical events (integration times of Newton's equations) during the lithiation and 1 fs during equilibration are more than enough to capture most of the relevant chemistry needed; however, the dendrite growth in a battery may take a few hours to form, simply because of the slow motion of the Li-ions, ∼1 mm per hour for a typical commercial battery. Thus, it really does not matter if we accelerate the rate of the important events by 10^10^ times faster or so; we will get the same results if we run the simulation by 10^10^ times longer. What we learn from these simulations can be used in future simulations and perhaps in coarse grained ones where the question of the dendrite growth-time may be posed.

To investigate the formation of dendrites, we use a protocol in which Li atoms under the surface of an electrode are neutral as being part of a Li-metal or LiSi phase; all Li atoms above the surface are Li^+^; and all atoms on the surface are negatively charged, representing the surface charge in the negative electrode. In this case, if a Li or a Li^+^ ends up above the Li^−^, then the negative charge is transferred to the Li or Li^+^ becoming Li^−^, or Li, respectively, and simultaneously the original Li^−^ is oxidized to Li below the surface. We re-assign these charge distributions every 10 fs. This protocol keeps the net charge of the whole system neutral and it emulates the polarization of the surface of the anode,^7,64^ since the lithium metal and the alloy SiLi should behave as electric conductors. Under this protocol, charges are carefully assigned to keep charge neutrality at all times. On the other hand, Li-ions are maintained at 1 M in the electrolyte. As ions are de-solved from the electrolyte and enter the anode, an equal number of ions comes out from the cathode and dissolves in the electrolyte; the electrolyte continues being at 1 M at all times. The charge equilibration mentioned above is performed to follow the nuclei dynamics and not the actual electron dynamics. 10 fs corresponds roughly to the period of the fastest vibrations in the box under analysis, *i.e.*, the C–H symmetric and antisymmetric stretchings, thus the 10 fs is sufficiently small enough to get a good estimate of charges for a given nuclei (ionic) conformation. Also notice that charges are valid only as a set and not as individually converged quantities. There is not an expectation value for atomic charges.^59^

### Simulations

**Fig. 2:**
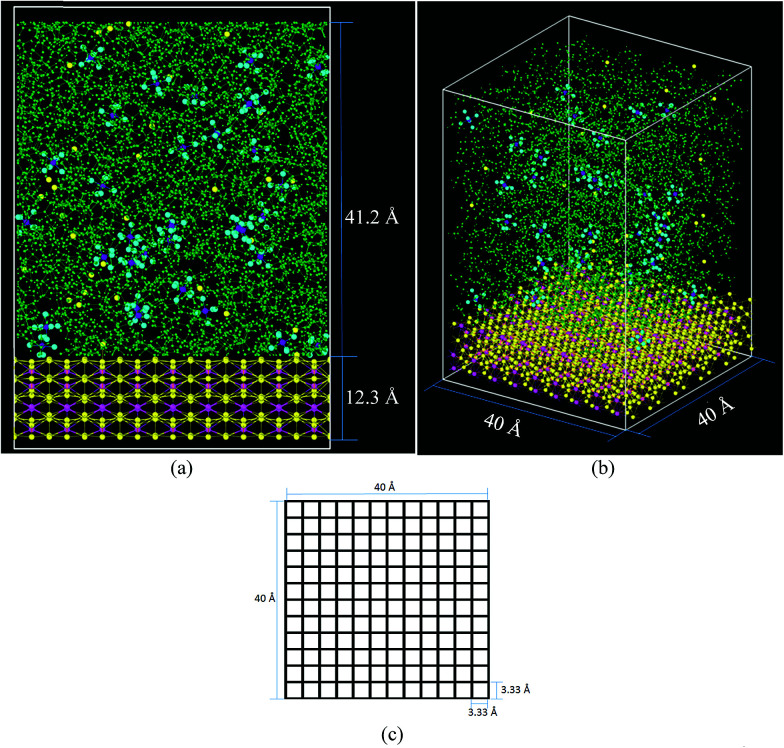
Initial simulation cell comprising 578 EC (green), 225 Si (pink), 945 Li (yellow) and 33 Li^+^–PF_6_^−^ pairs (yellow, purple, and light blue, respectively) (a) front view (b) orthogonal view, (c) basal plane divided in 12 × 12 = 144 square sectors.

The cell (Fig. 2) has a volume of 40 × 40 × 65 Å^3^. The bottom part (Fig. 2a and b) contains the lithiated silicon anode, Li_21_Si_5_, with a volume of 40 × 40 × 12.3 Å^3^. The electrolyte on top of the Li_21_Si_5_ anode (volume = 40 × 40 × 41.2 Å^3^) is composed of a 1 M solution of LiPF_6_ diluted in ethylene carbonate (EC). The electrolyte contains 578 molecules of EC and 33 of LiPF_6_, corresponding to a density of 1.28 g cm^−3^, a value consistent with the EC experimental density of 1.32 g cm^−3^.^65^ The lithiated anode contains 945 atoms of Li and 225 atoms of Si, corresponding to a density of 1.086 g cm^−3^ which is smaller than the experimental density of similar composition Li_22_Si_5_ crystal with a density of 1.181 g cm^−3^.^66^ The discrepancy is because the force field prefers a more relaxed geometry. The cell is divided in square sectors of 3.33 Å per side (Fig. 2c). For each sector, the *z*-coordinate (height) of each Li belonging to the anode is calculated at every time step and the one with the highest value is considered part of the surface if at least it is bonded to a neighbor Li or Si that also belongs to the anode block. The threshold distance for bonding was set to 3 Å, which corresponds to the metallic Li–Li distance of 3.04 Å in a Li-metal crystal.^67^ The Li atoms forming the surface have partial negative charges (Li^*δ*−^), while the Li under the surface remain neutral and the Li above the surface are Li^+^.

During charge, an electric field is applied to the battery. A typical rechargeable lithium-ion battery yields a voltage of 3.6 V, but charging the battery needs a little more; thus, the voltage applied between electrodes is roughly 3.8 V in commercial batteries. However, as in several atomistic MD simulations, external electric fields cannot distinguish the conductive nature of the electrolyte, making the potential to be uniformly distributed among all components in the electrolyte along the axial direction. This uniform distribution of potential gives the impression that the applied electrical fields in the simulation are several orders of magnitude higher than those applied in a real battery. However, locally their effect is exactly the same as the energy provided by both fields to the ions are identical; the local effect on the ions is what really matters. It seems odd that electric fields we have to use are in the order of 1 V Å^−1^; however, electric fields of such magnitude are very common at atomistic dimensions. Those are the required fields to apply to the ions in order to drift them from cathode to anode during charge. Thus, in a typical or commercial battery, it is the field that provides the ion the energy needed to go through the barriers the ion finds in its way, especially to react at the electrodes. It is not that in a real battery we are supplying such voltage per unit of length even when the ion is not there. A voltage drop per ion is supplied only when an ion is present and the voltage drop is practically zero wherever no ions are present. Just to clarify a little bit on the electrical fields that take place internally on a commercial battery, let us calculate the field of one ion on the location of the other ion at a distance *r*. This can be easily calculated in atomic units as *e*/*r*^2^, assuming *r* = 20 bohrs (∼10 Å), which is a very common separation between ions at 1 M. This yields an electric field of 2.57 V Å^−1^. Yes, this voltage is ∼10^7^ times bigger (!) than practical fields from a power supply in a commercial battery if the potential were distributed uniformly across the anode and cathode.

Following further this example, the energy of interaction between the two charges is just 1.34 eV, which is perfectly normal at atomistic dimensions, but producing a field that looks amazingly large. Thus, although the applied electric fields seem extremely large, they correspond with the energies needed by the ions to move and/or react in the battery. The high fields do not affect sites where no ions exist. Therefore, the electric field in the simulation, which acts along the whole battery in the direction from cathode (positive) to anode (negative), provides the correct amounts of energy to the ions so they can drift to the anode. Thus, the external field should yield the proper voltage or energy per ion. Classically, it is impossible so far to represent a conducting medium in an atomistic simulation, such that the applied voltage drops only or mostly at the ions. Voltage distributes uniformly in the whole electrolyte but affect only the ions. The internal fields are additive and are produced by the actual distribution of charges (densities) and by defects on the structure. Here we hope to resolve a strong misconception on this topic. Just to clarify further, the external field is the one produced by the power supply locally on the ions when charging a battery. It is the one that provides energy to the ions so they can drift though the solvent. Simply multiplying this field used in the simulations by the length between electrodes does not correspond to the actual applied external voltage in a commercial battery. In a real battery, the external voltage does not depend (in practical terms) on the length of the battery as the voltage drops practically only on the reaction sites and not uniformly along the whole length between cathode and anode.

We choose an electric field of 0.5 V Å^−1^, which, according to experience is a good compromise between accuracy and practicality.^56,68^ These simulations focus on the growth of dendrites on the anode; therefore, the cathode is simply an ion-emitter that maintains the rate of emissions low enough to avoid strong interactions between neighbor ions but fast enough to reduce simulation times to a minimum. The whole nanobattery was equilibrated before the application of electric fields. Under the application of electric fields, the charging was set at 300 K with a relaxation time of 10 fs, which seems to be a good compromise to follow the rapid dynamics of the Li-ions and considering the nonexistence of other destructive events in the battery as we are assuming dendrites formed in cracks already produced on the surface of SEI covering the anodes. These cracks certainly change the uniform charge distribution on the active regions of the anode. The nominal atomic charge is very small on the surface atoms of the electrodes; therefore, we decided to perform simulations with very different values of charge and spatial distributions. The first one corresponds to a constant charge of −1*e* in all atoms of the surface, left by the crack, representing the worst-case scenario for a nanoscopic region. The second case corresponds to a charge gradient where only the atoms at peaks have a charge of −1*e* but the charge decreases uniformly to zero as valleys are reached. These two cases have the principal purpose to check if these charges or distributions can produce lithium dendrites. The third and fourth cases are for a constant charge distribution of an equivalent capacitor of the same dimensions as our nanobattery (nBatt) and for an A&T commercial battery (A&T), respectively. Assuming the simplest battery model, a capacitor of capacity *C* = *Q*/*V* = (*ε*_r_ × *ε*_o_ × *A*)/*d*, where, *Q* = charge, *A* = area of the electrodes, *d* = separation between anode and cathode, *ε*_r_ = relative permittivity, *ε*_o_ = permittivity in vacuum, *ε*_r_(EC at 300 K) ≈ 94.7,^69^*ε*_o_ = 8.85 × 10^−12^, and *V* = 3.8 Volt, *d*(commercial battery) = *d*_A&T_ = 25 μm,^70,71^*d* = *d*_s_ = 41.2 Å, *A*(commercial battery) = *A*_A&T_ = 422 cm^2^,^70,71^*A*(nBatt) = *A*_s_ = 1600 Å^2^. Using these values, we get for the nBatt, *C* = 325.59 × 10^−20^, and *Q*_s_/*A*_s_ = 0.7733C m^−2^, yielding a charge per atom of −0.537*e* considering 144 atoms of Li^−^. On the other hand, for the commercial battery, *Q*_A&T_ = 5.375 μC and *Q*_A&T_/*A*_A&T_ = 1.273 × 10^−4^ Coulomb per m^2^. For the nBatt anode, charge per Li^−^ = −88μ*e* considering 144 atoms of Li^−^, thus the third and fourth cases correspond to superficial atomic charges of −0.537*e*, and −88μ*e*, respectively.

It is worth to mention that there is not a duplicative effect when assigning charges and applying external fields simultaneously. Internal fields created by the charges alone do not induce any drift of ions because of compensation by counterions in the neighboring atoms. The anomalous charge distributions we test are attributed to the crack formation on the surface of the solid electrolyte interphase (SEI), allowing the contact of the electrolyte solution with the anode material; in this case the anode was already an alloy of SiLi due to previous lithiation of the silicon anode. In addition, local electric fields due to interatomic effects are always there randomly distributed in magnitude and direction, even when the external field is zero.

## Results and discussion

### Equilibration and settings

**Fig. 3:**
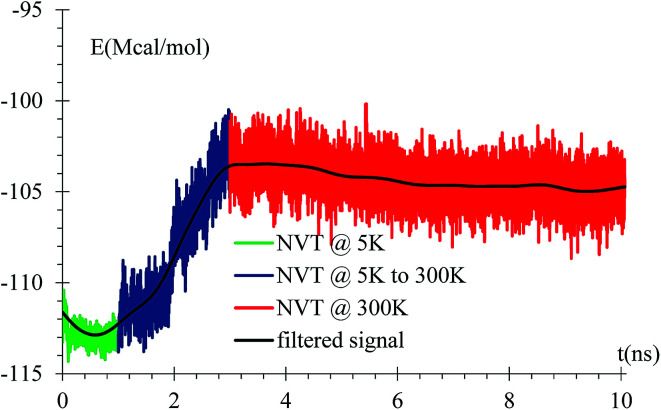
Energy *versus* time during equilibration stages at 5 K, rise from 5 K to 300 K, and at 300 K. The filtered curve is obtained by applying the discrete-time average function 10^5^ times, *E*_*t*_ = 1/4*E*_*t*−1_ + 1/2*E*_*t*_ + 1/4*E*_*t*+1_, where the time-intervals between samples correspond to 10^3^ simulation steps or 1 ps. This protocol is used in all following filtered curves, unless stated otherwise.

An equilibration at 5 K is performed for 1 ns, then the temperature is increased from 5 K to 300 K for 2 ns, and finally an equilibration at 300 K for 7 ns is done with a relaxation time of 10 fs. A NVT ensemble is used on the whole equilibration process (Fig. 3).

### Dendrite formation

**Fig. 4:**
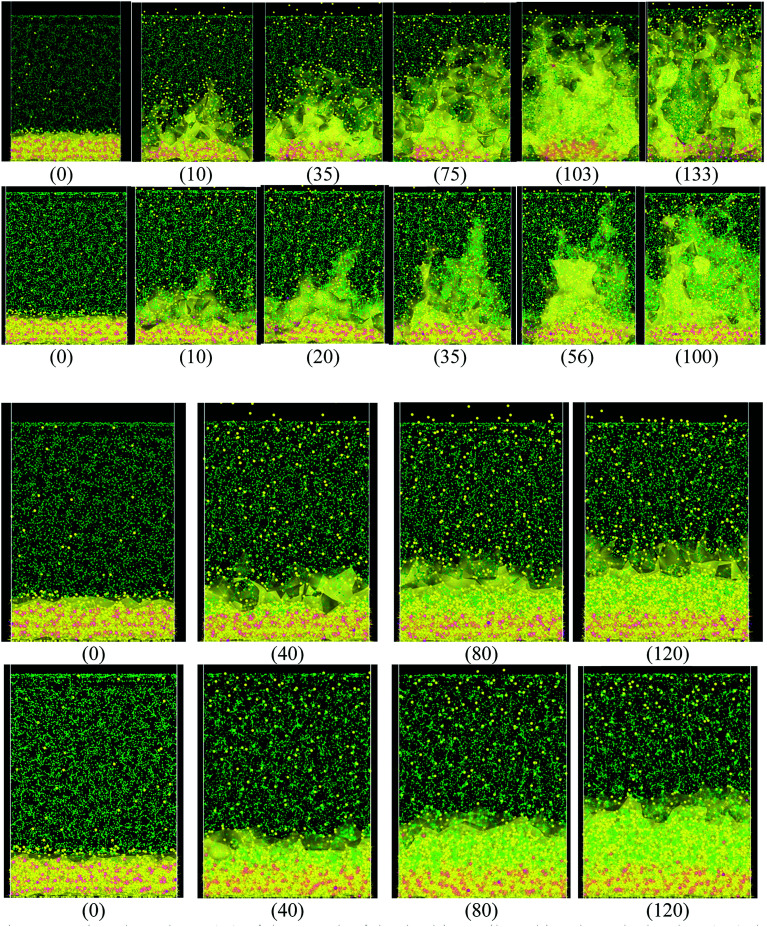
Time dependence (ps) of the growth of the dendrite until reaching the cathode when (top) the charge of the atoms on the surface is constant and equal to −1*e*, −0.54*e*, −88μ*e* respectively and (bottom) when the charge on the surface is variable according to the height. Li (yellow), Si (pink), EC and PF_6_^−^ (green). It can be observed in the case where the charge on the surface is constant, the dendrites are much more pronounced.

When the Li atoms on the surface have a charge of −1*e*, after a few ps from the beginning of the simulation, a dendrite can be observed with its height steadily increasing for 103 ps. At this time, the dendrite reaches the cathode, short-circuiting the nanobattery. When the surface Li atoms charge is −0.537*e*, the short circuit occurs at 56 ps. For the −88μ*e* charge, the dendrite formation is insignificant due to the low charge and short time of simulation, but an initial small dendrite formation is observed. However, when there is a charge gradient, the growth is more uniform in all sectors than for the case of constant charge (Fig. 4).

**Fig. 5:**
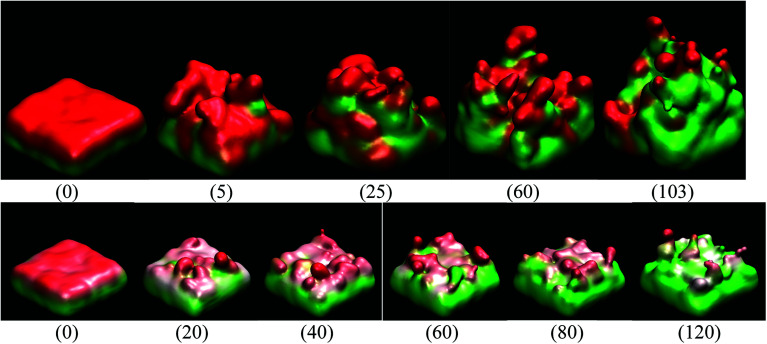
Time dependence (ps) of the surface charge when (top) charge on all surface atoms is −1*e* and (bottom) charge on the surface atoms is a function of height. Positive charge (red) and neutral charge (green).

Since we assume that the dendrite forms on a crack of square superficial shape, the surface under study is the top one shown in Fig. 5 at time 0. Thus, we are referring on the following only to this top surface. The surface always has a negative charge, while the rest of lithium and silicon underneath are neutral (Fig. 5). Fig. 5 shows only two cases of four, since cases 1, 3, and 4 have an iso-charge surface (Fig. 5 top) and only case 2 shows a charge gradient distribution on the surface (Fig. 5 bottom). Fig. 5 shows clearly at all times the charged zones; however, dendrites look less porous and hollow than they really are due to the integrative nature of the graphics software.

**Fig. 6:**
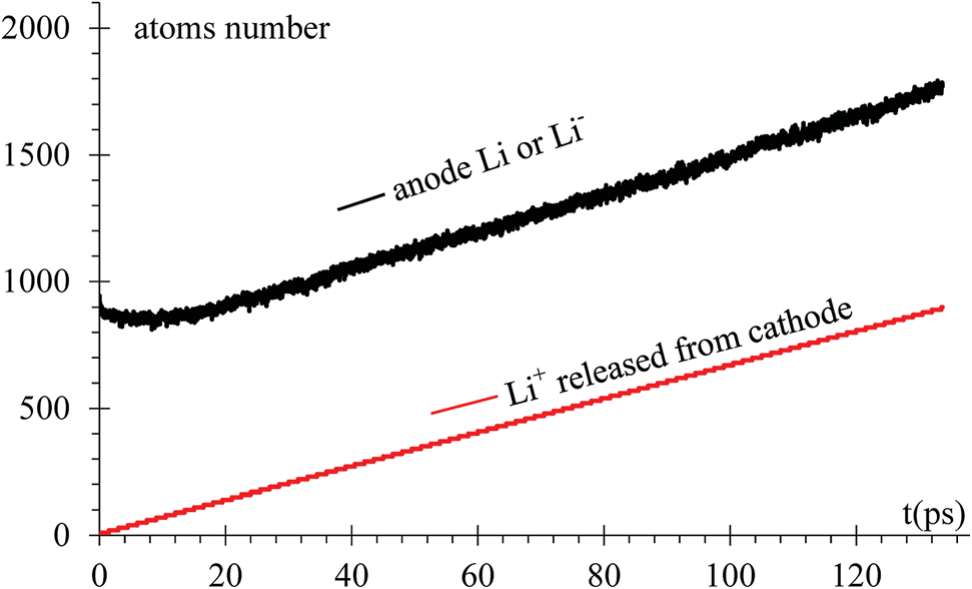
Li and Li-ions populations in the anode *versus* time.

As expected, after a transient of 8 to 10 ps, when the surface has a charge of −1*e*, the rate of growth of Li in the dendrite equals the rate of Li-ions emitted from the cathode, 6.66 ions per ps (Fig. 6). Since the Li surface is at a negative potential from to the power supply during charge, some of surface Li^*δ*−^ might be ejected by coulombic interactions. In turn, these negative ions swap to positive charge (oxidized) when another reduced neutral Li becomes negative (polarized) on the surface while the negative ion is above the surface of the LiSi alloy, and returns as positive ion (oxidized) to the surface; thus, there is some chance of particular ions enter in this redox loop and not being part, temporarily, of the LiSi alloy. The curve shows that the number of Li in the anode slightly decreases right after applying the electric field.

**Fig. 7:**
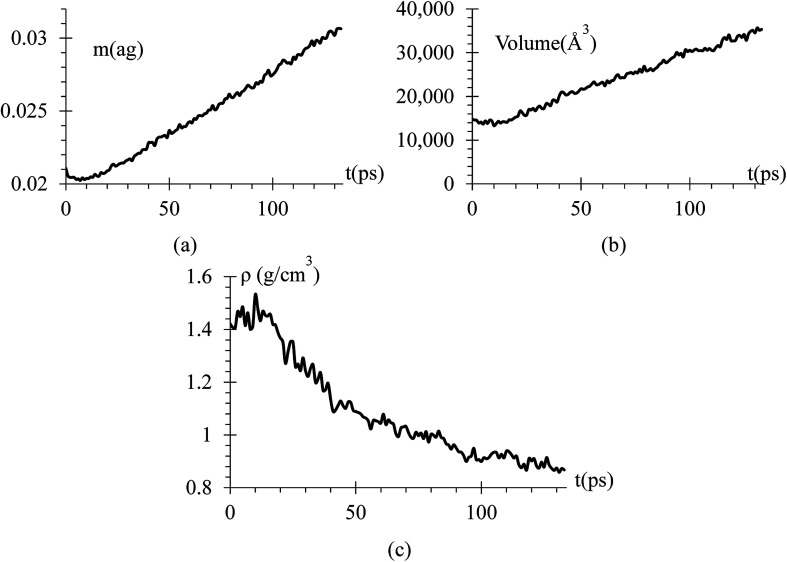
(a) Mass of the dendrite in attograms. (b) Volume of the dendrite using a convex hull algorithm, which do not consider the bond distance with the nearest neighbor atom; therefore, the volume is a little lower than it really is. (c) Density of the dendrite showing values between the silicon density of 2.329 g cm^−3^ and lithium metal density of 0.535 g cm^−3^.

Since we know the number of Li flowing from the cathode, and the number of Si and Li on the anode are constant, we can calculate the dendrite mass (Fig. 7a). Using this information, we calculate the volume and density of the dendrite (Fig. 7b and c) using the software OVITO^42^ that uses a convex hull algorithm.^72^ The dendrite density (Fig. 7c) shows decreasing values between 1.55 to 0.85 g cm^−3^, which are between the silicon and lithium densities of 2.329 and 0.535 g cm^−3^, respectively.

Observing the lithiation process, the average time between a Li^+^ emission from the cathode and its deposition on the surface is approximately 10 ps and since the density of lithium is 535 kg m^−3^ and its molar mass is 6.941 g mol^−1^, we can calculate the height of the LiSi alloy when their growth is uniform, *h* = *h*_i_ + *n*_Li_ × *V*_Li_/*A*, *h* = alloy height (Å), *h*_i_ = initial height = 12.3 Å, *n*_Li_ = number of new lithium in the dendrite, *V*_Li_ = volume of one lithium (Å^3^) = 6.941/(6.022 × 10^23^ × 535000) = 21.544 Å^3^, *A* = area of the electrodes = 1600 Å^2^, *h* = 12.3 Å + *n*_Li_ × 0.013465 Å.

**Fig. 8:**
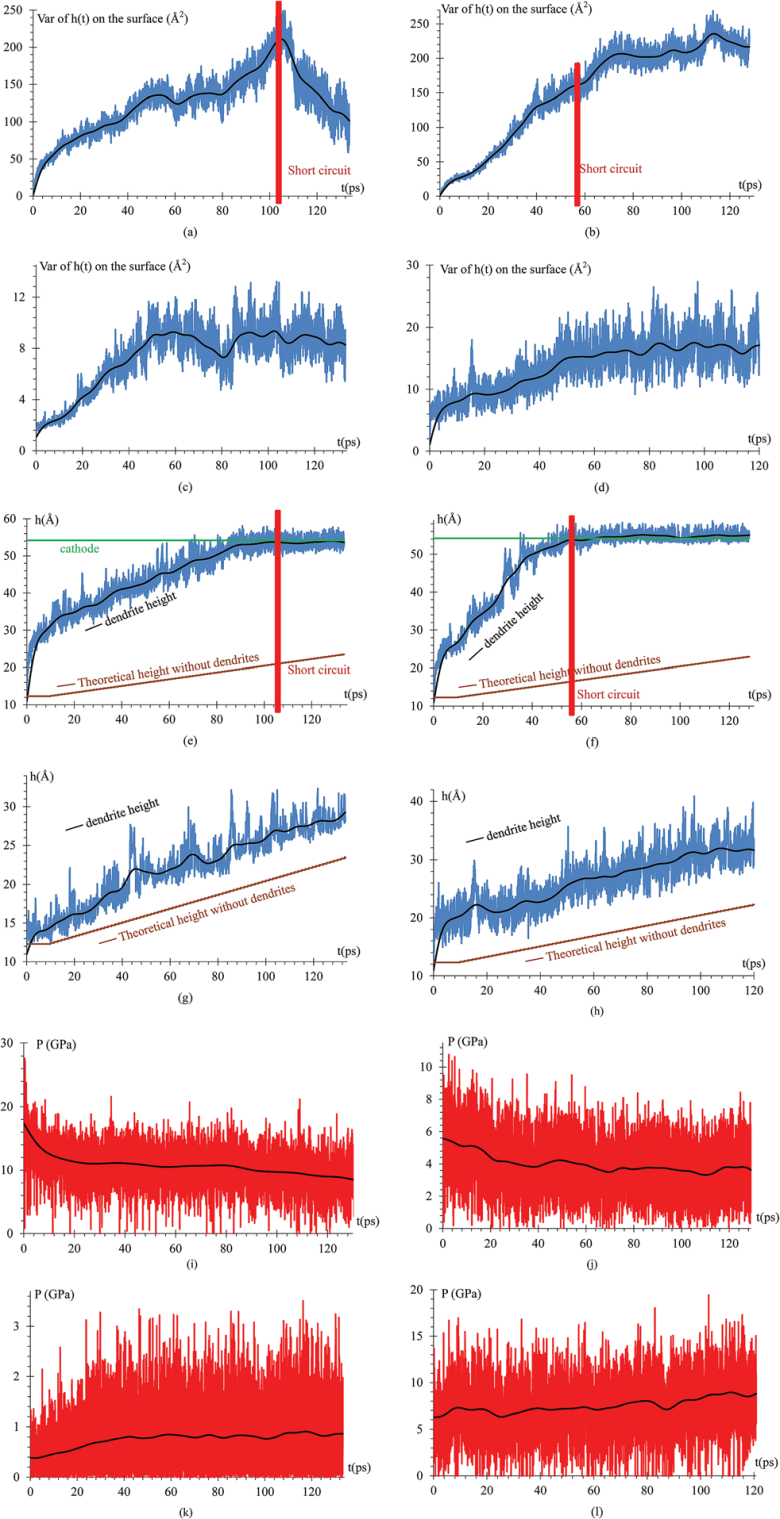
Variance height of atoms on the surface *versus* time when the charge on the atoms of the surface is (a) −1*e*. The variance starts to decrease at 103 ps because a short circuit is produced; the peak cannot grow further but the valley does and that decreases the variance. (b) −0.537*e*. The variance increases more than in any other case because the growth is less uniform, and the variance can continue growing after the short circuit. (c) −88μ*e*. The variance increases almost linearly up to roughly a steady value but at slow growth. Since this case is not as accelerated as the previous one, it would have to extend the simulation for larger times to see the results of the previous case when the charge in the atoms on the surface is −0.537*e* (d) distributed. The variance strongly increases but it is slow when compared with the cases in which the charge of the atoms on the surface is constant. No other cases for distributed charges is done since the other cases we tried were discouraging when compared to the cases when the surface atoms had constant charge. Height of the highest atom of the dendrite *versus* time, when the charge on the surface is (e) −1*e*. (f) −0.537*e*. (g) −88μ*e*. Due to the low charge, the lithium dendrite formation occurs on a smaller scale but still is clearly observable. (h) Distributed charges. (i) Dendrite growth pressure *versus* time, when the charge on the surface is −1*e*. In this case, the highest pressure is obtained, but since this case was performed only to verify the operation of this protocol, this value will not be considered as a reference of the pressure in a real case. (j) Dendrite growth pressure *versus* time, when the charge on the surface is −0.537*e*. This case obtained pressures close to 6 GPa, and since this case is a way to accelerate what would happen in a real battery this magnitude of pressures could happen in a real case. (k) Dendrite growth pressure *versus* time when the charge on the surface is −88μ*e*. This pressure is the smallest, but it is only the initial pressure that occurs when the dendrite starts to form; thus, this value between 0.4 GPa and 1 GPa can vary greatly if the simulation continues for much longer time. (l) Dendrite growth pressure *versus* time when the charge on the surface is not uniform.

To quantify the dendrite formation when the charges on the surface are −1*e*, −0.537*e*, −88μ*e* and distributed, we calculate the *z*-coordinate variance (height) of the Li or Si of the anode surface (Fig. 8a–d); thus, we calculate the maximum height of an atom in the dendrite (highest peak) and compare such height with that of the alloy when there are no lithium dendrites (Fig. 8e–h). The pressure on the surface in the *z*-axis is also calculated as the dendrite grows in this direction (Fig. 8i–l).

### Radial pair distribution function and bond vibrational frequency

**Fig. 9:**
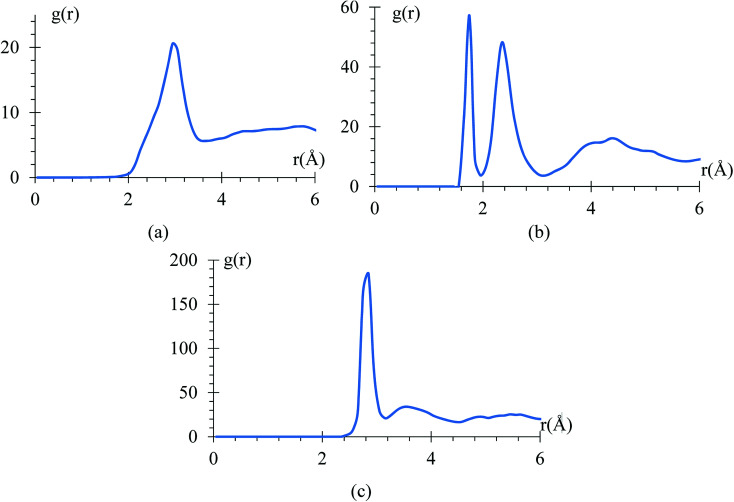
RDF of (a) Li–Li (b) Si–Li (c) Si–Si.

The radial pair distribution function (RDF), *g*(*r*), is calculated to check consistency of the geometry with the force field MEAM potential (Fig. 9). These graphs are calculated using the software VMD,^41^ for the case when the atomic charges on the surface of the LiSi alloy are −1*e*. The peaks show the equilibrium distances of Li–Li bond around 2.95 Å, which is similar to the experimental metal Li–Li distance of 3.0398 Å.^67^ These graphs correspond to the average of 7419 snapshots through the process of lithiation.

**Fig. 10:**
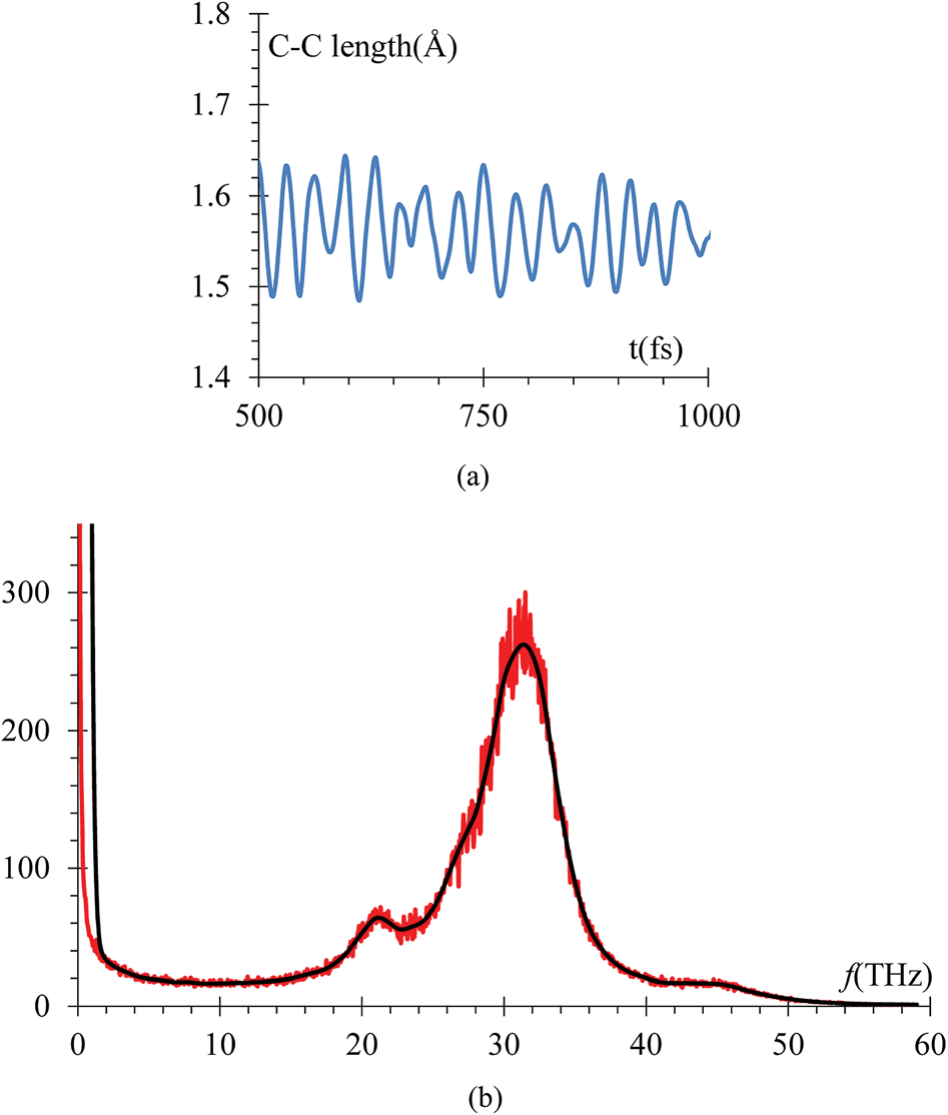
(a) Variations of a typical ethylene carbonate C–C bond *versus* time in the electrolyte solution after the equilibrium and immediately after starting the lithiation. The C–C bond average equilibrium distance is 1.53 Å. (b) Average of spectral analysis of all stretching C–C modes during the lithiation, yielding a frequency of 31.25 THz, corresponding to a wave number of 1025 cm^−1^. We reduced the low pass filter to only 100 passes for this particular case.

Another control run we choose to make sure the harmonic component of force field yields the correct C–C bond stretching frequency of an EC molecule is to compare it to the one obtained from *ab initio* calculations. The C–C distance oscillations *versus* time (Fig. 10a) in the electrolyte solution after the equilibrium and immediately after starting the lithiation yielded a C–C bond average distance of 1.53 Å in good agreement with usual values. Then, the C–C length signal is fast-Fourier transformed, obtaining the frequency spectrum shown in Fig. 10b. This spectral analysis yields a frequency of 31.25 THz (Fig. 10b), corresponding to a wave number of 1025 cm^−1^, which is also in good agreement with typical values for such vibration. We reduced the low pass filter to only 100 passes for this particular case as that was enough to provide a clear filtered signal.

The vibrational frequency calculated using DFT of the C–C bond in EC, is 29 THz, which compares with the average of spectral analysis of the average C–C bond stretches (Fig. 10) of the ethylene carbonate molecules from the electrolyte solution where the frequency peaks around 31.25 THz or 1025 cm^−1^, so the margin of error is less than 10% and can be attributed to the environment as the DFT calculation was performed in vacuum.

## Conclusions

Molecular dynamics simulations were used to analyse the lithium dendrite formation following a protocol for its growth. It was observed that the formation of lithium dendrites starts quickly and instantaneously after the solvated lithium has direct contact with the LiSi alloy. This is more intense in the case when the small surface of the crack is polarized with large negative charges. We also find that the dendrite growth rate after a few ps is maintained almost constant until moments before the short circuit. The lithium dendrites that formed under this protocol are very high and sharp (peak–valley distance). However, when the surface has a non-uniform negative charge, the growth is slower and more uniform. For this reason, we infer that the distribution of charges on the surface of a LiSi electrode, and especially, the concentration of charge in particular spots such as solid electrolyte interphase cracks, determines the dendrite formation. Dendrites created in this way are porous and hollow, characteristics that help a spontaneous and quick growth. Future work on this can certainly help us to define the cause of such uneven charge distributions and the possible routes to mitigate dendrite formation and growth by testing several other electrolytes and additives including solid electrolytes.^73,74^ Based on this work, electrolytes must resist pressures higher than 6 GPa to avoid dendrite formation without suffering structural damage.

This study does not include other large number of possible reactions that may take place before dendrites are formed. This study focuses on those in which Li^+^ still reduces at the anode in a battery that is working properly; however, it does not focus on cases when the battery fails for other reasons, such as gas production followed by an explosion or thermal runaway among several others. It is reasonable to think that it cannot be a MD study that covers all possible effects that may take place in a battery if we want to find out the reasons for the formation of dendrites. Certainly, if the formation of gases damages the battery, there is no reason to further study the anode for formation of dendrites. Also, we already reported the charging process for this type of anode in detail.^56^ Therefore, our present study focuses on the plating and conditions for the growth of dendrites of Li on the anode after the expansion of the anode cracks the SEI, ignoring those conditions that are not in the route to the production of dendrites. On the other hand, simulation and experimental relevant times cannot be compared. They are calculated/measured at very different scales. Dendrite growth may take a few hours to form simply because of the slow motion of the Li-ions, ∼1 mm per hour for a typical commercial battery. Note that we are not concluding or reporting the times of formation of dendrites; we are reporting possible conditions that will favor their formation.

At this point, we believe, our simulations will be more complementary rather than mutually corroborative with experiments. With our procedure we can study the effects of specific surface charge distributions or take surfaces from experiments caused by cracks due to the expansion of silicon electrodes and determine the dendrites that will be formed. Then, the predicted dendrites can be compared to those from theoretically guided experiments in order to rationalize behaviors. That, we believe, is the way how we can progress in this field. On the other hand, dendrites may appear practically on similar conditions used here, through a crack or any defect that allows accumulations of charge species followed by metallization; all these may happen on a crack of 1 or few nm. The key point is metallization. Although we use Si as the base material, dendrites may appear in any other type of material where the active material is exposed to the solvent. This could be the case of Si fully embedded in carbon where a nanocrack exposes Si to the solvent. This report will trigger several others in the community due to the practicality of our procedure to be used with any other type of materials and the learning from these simulations can be used in future simulations and perhaps in coarse grained ones where the question of the dendrite growth-time may be posed. In summary, we conclude that dendrites grow when the charge distribution on the surface of the exposed solid electrode to the liquid electrolyte are too large compared to the estimates for a commercial lithium-ion battery.

## Conflicts of interest

The authors declare that there is no conflict of interests regarding the publication of this article.

## Supplementary Material
